# Emotional Intelligence Buffers the Effects of Negative Emotions on Job Burnout in Nursing

**DOI:** 10.3389/fpsyg.2018.02649

**Published:** 2018-12-21

**Authors:** Dorota Daniela Szczygiel, Moïra Mikolajczak

**Affiliations:** ^1^Sopot Faculty of Psychology, SWPS University of Social Sciences and Humanities, Sopot, Poland; ^2^Department of Psychology, Université catholique de Louvain, Louvain-la-Neuve, Belgium

**Keywords:** emotional competence, anger, sadness, occupational stress, nurses

## Abstract

The study was designed to examine whether trait emotional intelligence would moderate the impact of negative emotions at work on job burnout. A total of 188 female nurses participated in this study and completed measures of trait affectivity, emotional intelligence, anger and sadness at work, and burnout. The results revealed significant and positive relationships between both types of negative emotions and burnout above and beyond demographics and the nurses’ trait affectivity. Importantly, the study demonstrated that trait emotional intelligence buffers the effects of negative emotions on burnout. Specifically, anger- and sadness-related emotions predicted greater burnout among nurses with low trait emotional intelligence but not among nurses with high trait emotional intelligence. These results suggest that emotional intelligence training could be implemented to prevent the adverse effect of negative emotions felt at work on job burnout.

## Introduction

Job burnout is a specific disorder resulting from prolonged exposure to high job demands in the absence of enough resources to compensate for their effects (Demerouti et al., 2000; Maslach et al., 2001; Bakker et al., 2004, 2014; Hu et al., 2017). There is ample evidence showing that burnout is costly for both individuals and organizations (for a review, see Cordes and Dougherthy, 1993; Swider and Zimmerman, 2010): it affects workers’ well-being, decreases job performance, and increases absenteeism and the intention to leave the job. The majority of research on burnout has been conducted in the human services (Schaufeli and Enzmann, 1998) and nursing professions have been described as particularly susceptible to burnout (Demerouti et al., 2000; Maslach et al., 2001; Consiglio, 2014; Munnangi et al., 2018). In the United States, up to 45% of nurses working in hospitals reach high burnout scores (Aiken et al., 2001).

Burnout has been shown to have a deleterious impact on nurses, as it affects their health (e.g., Shimizu et al., 2005; Jaworek et al., 2010; Duan-Porter et al., 2018). It also affects healthcare organizations, by increasing absenteeism (Iverson et al., 1998), job dissatisfaction (Wolpin et al., 1991) and intention to leave the profession (Leiter and Maslach, 2009; Heinen et al., 2013). Last but not least, burnout affects patient safety: higher rates of burnout among healthcare professionals are associated with lower quality of care (Poghosyan et al., 2010), increased frequency of neglectful behaviors toward patients (Reader and Gillespie, 2013) and increased frequency of adverse patient events, such as nosocomial infections (Cimiotti et al., 2012) and medication errors (Tsiga et al., 2017).

Nurses are exposed to a variety of occupational stressors, ranging from organizational factors such as heavy workloads and time pressure (Demerouti et al., 2000), to interpersonal conflicts at work (Dåderman and Basinska, 2016) and patient-related factors such as the suffering of or verbal aggression from patients and their relatives (Edward et al., 2014; Viotti et al., 2015). These conditions make nurses particularly vulnerable not only to stress but also to the experience of negative emotions (NE) (e.g., Acquadro Maran et al., 2018). Despite the significant role that emotions play in nursing practice (Bulmer-Smith et al., 2009), surprisingly little attention has been paid to the NE–burnout relationship and even less to the moderators of this relationship. These are the issues that inspired the current study.

The few studies that have measured both NE and burnout provide evidence suggesting that NE felt at work are related to burnout among nurses. Hillhouse and Adler (1997) were the first to demonstrate a statistical association between NE and burnout in nurses. In a cross-sectional study on 260 hospital nurses, they showed that nurses with the highest levels of anger and depression experience at work also reported the highest burnout scores. Erickson and Grove (2007) corroborated this relationship in another cross-sectional study on 829 nurses: younger nurses (i.e., under 30 years of age) with higher levels of anger and frustration at work reported higher rates of burnout. Similar results were obtained by Ersoy-Kart (2009), who conducted a study in a sample of 100 nurses (of which 47 worked in the private sector and 53 in the public sector) and observed that expression of anger at workplace correlated positively with emotional exhaustion scores. Zellars et al. (2004) demonstrated in a study on 296 hospital nurses that negative moods (states) at work correlated positively with emotional exhaustion, depersonalization, and reduced personal accomplishment. Importantly, in the study of Zellars et al. (2004), emotional exhaustion and depersonalization were predicted by negative moods over and above affectively saturated personality dimensions, such as neuroticism and extraversion. Finally, in the most recent study, Barr (2018) showed positive correlation between state negative affect and burnout in 142 nurses. It should be noted that the NE-burnout relationship was also observed among employees working in other than the health service sector. For example, Basińska et al. (2014) demonstrated positive correlations between burnout and NE (e.g., anger, boredom) in police officers. Similarly, Bedyńska and Żołnierczyk-Zreda (2015) observed positive association between NE and burnout in a study among managers, administrative staff, and shop assistants.

The foregoing studies constitute important evidence that NE could indeed contribute to nurses’ burnout. In all above-mentioned studies, however, emotions were measured only once, which raises the possibility that the relationship between NE and burnout was driven by the momentary affective state. Moreover, respondents’ emotions were assessed using the Positive and Negative Affect Schedule (PANAS; Watson et al., 1988) (see the study of Barr’s, 2018), the Job Affect Scale (JAS; Brief et al., 1988) (see Zellars et al., 2004), and the Profile of Mood States (POMS; McNair et al., 1981) (see Hillhouse and Adler, 1997), thus providing a global NE score, but not referring to specific (discrete) emotions at work. Moreover, nurses’ affective dispositions were not controlled for [with the exception of the Zellars’s et al. (2004) study], which limits the interpretation of the findings (i.e., the correlation between NE and reported burnout could simply be the product of a third variable, such as trait negative affectivity).

Given the importance of this issue, the current study aims to extend previous findings in four ways: first, by re-examining the association between NE and burnout using daily reports on emotions, collected over five consecutive days; second, by examining whether specific (discrete) NE, such as sadness-related and anger-related emotions uniquely contribute to nurses’ burnout; third, by investigating whether sadness- and anger-related emotions contribute to burnout beyond the respondents’ trait positive and negative affectivity; and, fourth, because we believe that attention should be paid to the factors that may alleviate the adverse effect of NE on job burnout, we aimed to examine whether the strength of the association between NE and burnout varies according to each nurse’s emotional intelligence (EI).

### The Role of Trait Emotional Intelligence in the Negative Emotions–Burnout Relationship

Do negative emotions always lead to burnout? The answer to this question requires the examination of emotion-related individual differences. The construct widely used to account for these differences is EI (Petrides and Furnham, 2000, 2001; Petrides, 2011). Although the past decade has witnessed an abundance of theoretical and empirical work dealing with EI in the nursing profession (e.g., Gerits et al., 2005; Kooker et al., 2007; Landa et al., 2008; Quoidbach and Hansenne, 2009; Kozlowski et al., 2017; Snowden et al., 2018), knowledge about the role of EI in nursing is still limited (Bulmer-Smith et al., 2009; Petrides and Sevdalis, 2010; McCloughen and Foster, 2018).

The notion of EI aims to capture individual differences in the way people process emotions and, in particular, in the way in which they identify, express, understand, regulate and use their emotions and those of others. Individuals high in EI are able to identify their own emotions and emotions of others, they are able express emotions in a socially acceptable manner, understand causes and consequences of emotions, use them to enhance their thoughts, actions, and social relations, and regulate them when they are not appropriate to either their goals or the situational context (Mayer et al., 2016; Petrides et al., 2016).

There are two main conceptualizations of EI: ability models (e.g., Mayer and Salovey, 1997) and trait models (e.g., Petrides and Furnham, 2003; Petrides, 2011), which have led to different lines of research and to some debates on the status of EI as a set of traits (best assessed via personality-like tests) or abilities (best assessed via intelligence-like tests). These debates between trait and ability conceptions of EI have resulted in an integrative model encompassing three levels: knowledge, abilities, and traits (Mikolajczak, 2009). The knowledge level refers to what people know about emotions and emotionally intelligent behaviors (e.g., *do I know* which emotional expressions are constructive in a given social situation?). The ability level refers to the ability to apply this knowledge in a real-world situation (e.g., *am I able* to express anger constructively in a given social situation?). The focus here is not on what people know but on what they can do: even though many people know that they should not shout when angry, many are simply unable to contain themselves. The trait level refers to emotion-related dispositions and captures people’s disposition to behave in a certain way in emotional situations (e.g., when I am angry, do I *typically* express my anger constructively?). As the foregoing illustrations should have made obvious, these three levels of emotion-related individual differences are loosely connected (Lumley et al., 2005; Cardoso-Seixas, 2016). In the current paper, we refer to the trait level because we are interested in what a person actually does, and how people typically behave in emotional situations.

Previous research has shown that trait EI was negatively associated with burnout in nursing and medical staff, in both cross-sectional (e.g., Weng et al., 2011) and longitudinal (e.g., Mikolajczak et al., 2007a) studies. Experimental studies confirmed that trait EI is causally involved in this relationship: when trait EI is increased through training, burnout symptoms decrease (Karahan and Yalçin, 2009). Gerits et al. (2005) observed, in a 2-year longitudinal study, that female nurses relatively high in EI declared less burnout symptoms than their low-trait-EI counterparts. There is also evidence that high trait EI not only mitigates the symptoms of burnout, but also mediates the relationship between burnout and organizational outcomes, such as turnover intention and job performance (Magnano et al., 2017).

The processes, however, which underline this protective effect of EI on burnout in nurses have not received much attention. Görgens-Ekermans and Brand (2012) provided evidence that trait EI could moderate the relationship between stress and burnout. This is not surprising as trait EI promotes better management of negative or stressful situations. Research shows that individuals high in trait EI are both more likely to appraise stressful situations as a challenge rather than a threat, and they are more confident that they can cope with such situations (Mikolajczak and Luminet, 2008). This results in significantly lower reactivity to stressful events at both psychological (i.e., mood deterioration) and physiological (i.e., salivary cortisol) levels (Mikolajczak et al., 2007a,b). Moreover, trait EI is associated with the use of more efficient emotion-regulation strategies (for a meta-analysis, see Peña-Sarrionandia et al., 2015), which should help nurses with high EI to decrease efficiently the intensity and duration of NE, thereby protecting them against burnout. Trait EI, however, does not only promote better management of stressful situations. It also promotes better management of anger and sadness (Mikolajczak et al., 2008). The current study builds on these findings and suggests that EI could also moderate the relationship between NE and burnout.

### The Current Study

In this study, we examined the impact of two state NE on burnout: anger-related emotions (ARE) and sadness-related emotions (SRE). We focused on these two state NE based on the results of our pilot study (see section “Materials and Methods”), which sought to identify the emotions most frequently experienced by nurses at work. We hypothesized that both ARE and SRE would be related to burnout. Examining this, requires to control for trait negative affectivity (NA), as high trait NA is associated with higher levels of burnout (e.g., Wright and Cropanzano, 1998) and increases emotional reactivity to negative-mood induction (e.g., Larsen and Ketelaar, 1991). This raises the possibility that NE are only spuriously associated with burnout, the actual “driver” of this relationship being trait NA (cf. Wright and Cropanzano, 1998). We, therefore, controlled for this possibility. If state NE are indeed associated with burnout (beyond trait NA), we predicted that this relationship would be moderated by EI: nurses high in trait EI should be better able to regulate NE and, therefore, experience lower burnout levels than their low-trait-EI counterparts. In summary, we propose the following hypotheses:

Hypothesis 1: State anger-related emotions experienced by nurses at work are positively related to burnout, beyond trait negative affectivity.
Hypothesis 2: State sadness-related emotions experienced by nurses at work are positively related to burnout, beyond trait negative affectivity.
Hypothesis 3: Trait emotional intelligence moderates the relationships between anger-related emotions and burnout in such a way that this relationship is weaker among those with higher emotional intelligence than among those with lower emotional intelligence.
Hypothesis 4: Trait emotional intelligence moderates the relationships between sadness-related emotions and burnout in such a way that this relationship is weaker among those with higher emotional intelligence than among those with lower emotional intelligence.

## Materials and Methods

### Participants

A total of 188 female nurses from three hospitals located in northern Poland participated in this study. The inclusion criteria for this study were as follows: voluntary participation, working with adults and working in shifts. The exclusion criteria: unwillingness to participate in this study and returning incomplete questionnaires. A total of 275 individuals initially expressed interest in this project, of which 188 actually participated (68%). Eighty-seven participants were excluded from the final sample, as they did not complete the questionnaires fully. The study involved nurses representing various care units: cardiology, surgery, orthopedics, internal medicine as well as anaesthesiology and intensive care. The participants were, on average, 42 years old (*SD* = 9.43), and ranged from 23 to 61 years old. The average number of hours at work/per week was 45 (*SD* = 8.11).

### Measures

#### Emotions

The emotions experienced by nurses at work were assessed using the Nurses Job Emotions Scale (NJES), which was created on the basis of a pilot study. We decided to create the NJES for two reasons. First, we were interested in measuring specific (discrete) NE and not just NE. Second, to the best of our knowledge, there is no established and published instrument for assessing self-reported discrete emotions experienced by nurses in the workplace. Therefore, prior to the study, we conducted interviews with 47 nurses, who were asked to name the emotions they most frequently experienced during an average working day. All of the nurses reported experiencing stress and a number of discrete emotions. After the exclusion of repeated and synonymous terms, we devised a list of ten emotions that are most frequently experienced by nurses at work. In descending order of occurrence frequency, nurses reported experiencing positive emotions of enthusiasm, joy, pride, and contentment. The negative they experienced were (again, in descending order of frequency) anger, irritation, sadness, disappointment, embitterment, and depression. Therefore, the NJES that was used in this study consists of these ten adjectives describing emotions. Participants rated the extent to which they felt each emotion at work. They were asked to fill the questionnaire in relation to the current workday, i.e., “How do you feel today?” The NJES was completed five times, i.e., once a day, over five consecutive workdays. The response options ranged from one (not at all) to five (very much). As recommended by Fisher (2000), we calculated the mean level of intensity of each of the ten emotions over 5 days, by averaging participants’ ratings on each emotion; for similar approach see also Conway and Briner (2002) and Grandey et al. (2002). Next, in order to examine the factor structure of the ten emotion items used in the NJES, a principal component analysis with oblimin rotation has been conducted. Three factors were extracted based on the eigenvalues-greater-than-one rule, explaining 79.61% of common variance and with a clear differentiation between three factors. The first factor accounted for 39.53% of the total variance and consisted of SRE (“depression,” “disappointment,” and “sadness”). The second factor accounted for 21.53% of the total variance and consisted of the four positive emotions (“enthusiasm,” “happiness,” “contentment,” and “pride”). The third factor accounted for a further 18.55% of the total variance and consisted of ARE (“irritation,” “embitterment,” and “anger”). All factor loadings exceeded 0.78. Finally, scores for anger-related and sadness-related emotions were created by averaging their respective items. Consequently, anger-related and sadness-related scores reflected the average intensity level of each type of emotions (i.e., ARE and SRE) felt by nurses within five working days.

#### Burnout

Burnout was measured with the Oldenburg Burnout Inventory (OLBI; Demerouti et al., 2010; Polish version by Baka and Basińska, 2016). The OLBI consists of 16 items, eight of which measure the exhaustion dimension of burnout, while the remaining eight measure the disengagement dimension of burnout. Items were scored on a four-point rating scale, ranging from one (strongly agree) to four (strongly disagree). Examples of items are: “There are days when I feel tired before I arrive at work” (reversed) and “it happens more and more often that I talk about my work in a negative way” (reversed), for exhaustion and disengagement, respectively. Scale scores were calculated by averaging the responses to the items associated with each burnout dimension, after appropriate items were reversed.

#### Trait Emotional Intelligence

Trait EI was assessed with the Trait Emotional Intelligence Questionnaire-Short Form (TEIQue-SF, Petrides and Furnham, 2006; Polish version by Szczygieł et al., 2015). This instrument is derived from the full form of the TEIQue (for an extensive description of the factors and subscales, see Petrides, 2011). The TEIQue-SF consists of 30 items with answers on a seven-point Likert scale ranging from one (completely disagree) to seven (completely agree). Examples of items are: “Expressing my emotions with words is not a problem for me” and “I often find it difficult to see things from another person’s viewpoint” (reversed). Scores for the TEIQue-SF were calculated by averaging the responses to the items, after appropriate items were reversed.

#### Dispositional Affectivity

Trait negative affectivity was measured using the Positive Affectivity Negative Affectivity Schedule (PANAS, Watson et al., 1988; Polish adaptation by Brzozowski, 2010). PANAS is a 20-item scale, which consists of 10 positive (e.g., excited, enthusiastic) and 10 negative (e.g., nervous, scared) adjectives describing emotional states. Participants were asked, “To what extent do you generally feel this way, on average, across all situations?” Items were scored on a five-point rating scale, ranging from one (very slightly or not at all) to five (extremely). Scores for negative and positive affectivity scale were formed by summing the responses to the appropriate items.

### Procedure

Participants were recruited by psychology students who volunteered to take part in this project. First, the purpose of the study and its voluntary nature of participation were explained to the supervisors of the nursing wards. The study protocol was reviewed and approved by the supervisors. Next, participants were asked face-to-face to participate in the study and were assured that their data would be kept confidential. Nurses who gave their informed consent to participate started by completing questionnaires on demographics and job characteristics, trait EI, trait affectivity and emotions at work (i.e., the NJES was completed for the first time); they were also asked to create their own “pseudo-code” (to ensure the anonymity of the study). They also received four envelopes with the numbers “first,” “second,” “third,” and “fourth.” The envelopes contained questionnaires for four consecutive days of the study. From the following day onward, they completed the questionnaire about emotions once a day, over four consecutive working days. On the last day, along with a questionnaire about emotions, the nurses filled out the burnout inventory. Questionnaires were filled in during coffee or lunch breaks. When we were designing this study, we planned to contact participants on each day of the study (via mobile phone short message service) to remind them to complete the questionnaires, but two hospitals did not allow us to so (to prevent nurses’ distractions during service). We were allowed to contact participants only at the beginning and at end of the study (and not every day). Therefore, the sealed envelopes were collected from participants on the fifth, sixth, or seventh day counting from the first day of the study (by the same student of psychology who initiated the study). This procedure has been applied to all participants.

Participants did not receive any compensation for participation in the study. Data were collected in January and February 2018.

## Results

### Preliminary Results

Table 1 contains the means, standard deviations, internal consistency coefficients (Cronbach’s α) and intercorrelations of all the variables measured. The pattern of correlations between the variables was in line with our expectations. Both ARE and SRE were significantly and positively associated with burnout. Trait EI was significantly and negatively correlated with burnout, and with both ARE and SRE. Age was negatively correlated, whereas intensity of patient contact was positively correlated with burnout.

**Table 1:** Internal-consistency reliability (Cronbach’s α), means, standard deviations, and intercorrelations among all study variables.

Variable	*M*	*SD*	1	2	3	4	5	6	7	8	Patient contact/day (%)	Age
1. Burnout	2.40	0.68	(0.92)								0.15*	-0.16*
2. Exhaustion	2.48	0.74	0.94***	(0.88)							0.08	-0.12
3. Disengagement	2.26	0.71	0.93***	0.74***	(0.86)						0.20**	-0.17*
4. Anger-related emotions	2.30	0.88	0.33***	0.30***	0.32***	(0.88)					0.08	-0.03
5. Sadness-related emotions	2.13	0.95	0.32***	0.25***	0.36***	0.28***	(0.95)				0.15*	-0.09
6. Emotional intelligence	4.65	0.89	-0.36***	-0.30***	-0.36***	-0.36***	-0.33***	(0.91)			-0.06	0.12
7. Trait negative affectivity	20.34	7.01	0.32**	0.30***	0.31***	0.43***	0.21**	-0.33***	(0.90)		0.05	-0.06
8. Trait positive affectivity	32.49	6.90	-0.15*	-0.09	-0.18*	-0.13	-0.11	0.29***	-0.27***	(0.84)	-0.04	0.06

*Diagonal values are the internal consistency estimates for each scale. ^∗^p < 0.05, ^∗∗^p < 0.01, ^∗∗∗^p < 0.001 (all two-tailed significance tests).*

### Main Results

In order to examine the main and interactive effects of NE (both ARE and SRE) and trait EI on burnout, a moderated hierarchical regression analysis was performed. The variables were entered into the regression model as follows: socio-demographic characteristics (nurses’ age and the amount of time they spent with their patients during an average working day) and both NA and PA were entered in the first step as control variables. NE (both ARE and SRE) were entered in the second step, and trait EI in the third step, in order to examine their unique contribution to the prediction of burnout. In the last step, in order to investigate whether the main effects of ARE and SRE were moderated by trait EI, two interaction terms were introduced, which were products of NE and trait EI. These terms were: ARE X trait EI; and, SRE X trait EI. Prior to creating interaction terms, NE and trait EI were centered, rendering the beta-weight of the interaction terms more interpretable (Cohen et al., 2003). When the interaction terms reached significant value, the simple slope procedure was employed to examine further the shape of the interaction (Aiken and West, 1991). The data were also analyzed for multicollinearity between independent variables using tolerance and the variance inflation factor (VIF). The results showed that there was no concern for multicollinearity in any of the regression models: all VIFs were below 2.5 (e.g., O’Brien, 2007). All statistical analyses were executed using the SPSS version 24 statistical package. The results of the regression analysis are depicted in Table 2.

**Table 2:** Results of moderated hierarchical regression analyses of negative emotions experienced by nurses at work and trait emotional intelligence on burnout.

Variables	Step 1 ß	Step 2 ß	Step 3 ß	Step 4 ß
Age	-0.11	-0.10	-0.09	-0.09
Patient contact (%/day)	0.10	0.06	0.07	0.06
Trait negative affectivity	0.30***	0.18*	0.16*	0.14
Trait positive affectivity	-0.06	-0.04	-0.01	-0.01
Anger-related emotions (ARE)		0.18*	0.14	0.09
Sadness-related emotions (SRE)		0.21**	0.17*	0.10
Emotional intelligence (EI)			-0.18*	-0.14
ARE X EI				-0.16*
SRE X EI				-0.16*
*R*^2^ (adjusted)	0.12	0.19	0.21	0.26
*ΔR*^2^	0.12	0.08	0.02	0.05

*^∗^p < 0.05, ^∗∗^p < 0.01, ^∗∗∗^p < 0.001.*

As depicted in Table 2, the full model explains 26% of the variance in burnout. Among the variables entered in the first step, only trait NA emerged as a significant predictor of burnout: higher trait NA was associated with higher burnout. In the second step, when ARE and SRE were introduced into the regression equation, the amount of variance explained increased significantly (*ΔR*^2^ = 0.08, *p* < 0.001). Trait NA remained significant, and both ARE and SRE were positively related to burnout beyond the control variables, which supports our 1a and 1b hypotheses. When trait EI was entered in the third step, the amount of variance explained increased significantly (*ΔR*^2^ = 0.02, *p* < 0.05). Trait EI emerged as a significant predictor of burnout: higher trait EI was associated with lower burnout. Both NA and SRE remained significant, but the correlation between ARE and burnout failed to reach the conventional level of significance (*p* = 0.06). Finally, when interaction terms were entered in the last step, two expected interactions between NE and trait EI were significant and the amount of variance explained increased significantly (*ΔR*^2^ = 0.05, *p* < 0.01).

**FIGURE 1:**
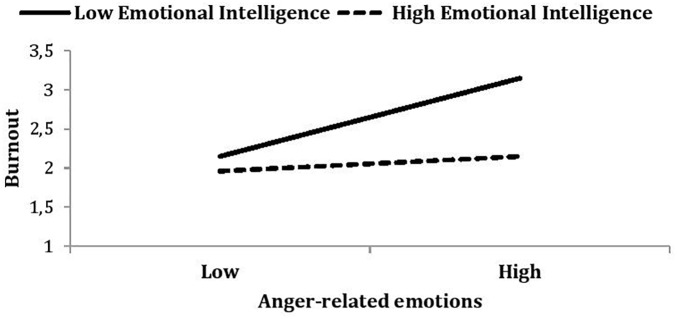
Burnout as a function of anger-related emotions (ARE) and emotional intelligence. Low ARE is defined as a mean – 1 standard deviation from the mean; high ARE is defined as a mean + 1 standard deviation. Note that this high/low split is for illustrative purposes only; the moderation analyses conducted use all variables as continuous variables.

Simple slopes analyses clarified the nature of these interactions. The relationship between ARE and burnout was plotted in order to compare nurses who scored more than one standard deviation above the average level of EI. As depicted in Figure 1, ARE predicted greater burnout among low-trait-EI nurses (β = 0.56, *p* < 0.01) but not among high-trait-EI nurses (β = 0.15, *p* = 0.37).

**FIGURE 2:**
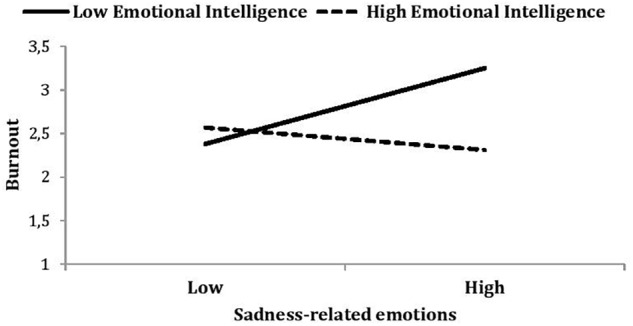
Burnout as a function of sadness-related emotions (SRE) and emotional intelligence. Low SRE is defined as a mean – 1 standard deviation from the mean; high SRE is defined as a mean + 1 standard deviation. Note that this high/low split is for illustrative purposes only; the moderation analyses conducted use all variables as continuous variables.

The relationship between SRE and burnout was plotted in a similar way. As shown in Figure 2, SRE predicted greater burnout among nurses low in trait EI (β = 0.66, *p* < 0.001) but not among nurses high in trait EI (β = -0.08, *p* = 0.65). In other words, NE experienced at work only increase burnout for nurses low in trait EI. Both H2a and H2b were supported. We repeated the above analysis for both exhaustion and disengagement as the dependent variable and we found a similar pattern of results (i.e., the shape of interactions) for each burnout dimension.

## Discussion

The present study sheds light on an important, yet under-researched, consequence of NE experienced by nurses at work: burnout. The results demonstrated a significant and positive relationship between nurses’ NE (both ARE and SRE) and burnout. These results substantiate previous research (Hillhouse and Adler, 1997; Erickson and Grove, 2007; Ersoy-Kart, 2009; Barr, 2018) and extend it by demonstrating that NE predict burnout above and beyond demographics and the nurses’ trait affectivity. This indicates the unique contribution of nurses’ NE in predicting burnout, which confirms our hypotheses and complements the aforementioned cited research of Zellars et al. (2004), who observed that NE predicted emotional exhaustion and depersonalization over neuroticism and extraversion.

There are at least two explanations as to why NE contribute to burnout: one is general in nature, while the second is specific for occupations involving intense interpersonal contact (Mikolajczak et al., 2007a). First, experiencing NE enhances one’s level of physiological and psychological arousal, which, if long-drawn, can have a deleterious effect on affective and cognitive functioning (e.g., Schröder, 1995; Szczygieł et al., 2012) and both mental and health (Lazarus and Cohen-Charash, 2001; Gross et al., 2011). Second, experiencing NE creates a specific burden upon nurses who, despite their true feelings, must maintain professional and supportive demeanours. Nurses are expected to express positive emotions (e.g., empathy and compassion), and hide NE (e.g., anger and resentment) (Diefendorff et al., 2011). Thus, in many job situations nurses must conceal their true emotional reactions and express emotions that they do not feel (Diefendorff et al., 2011), which leads to emotional dissonance and feelings of inauthenticity, both of which are considered significant occupational stressors leading to burnout (Hülsheger and Schewe, 2011).

Importantly, our findings show that NE do not *always* lead to burnout, but that they particularly do for nurses who lack EI. Coupled with the previous findings showing that EI reduces burnout symptoms (as previously shown by Gerits et al., 2005; Görgens-Ekermans and Brand, 2012; see also Mikolajczak et al., 2007a), with causality demonstrated experimentally by Karahan and Yalçin (2009), and by additionally showing that EI mitigates the effect of NE on burnout, the results of the present study bear several practical implications. Situations causing sadness and anger are an unavoidable part of nursing, and nurses should not, of course, be encouraged to become emotionally detached robots, therefore, healthcare organizations may want to consider providing EI training for their employees to help them strengthen their emotional skills. Such training would primarily aim to reduce the intensity, frequency, and duration of NE experienced at work and thus could likely prevent the adverse manifestations of NE, such as job burnout studied here.

Trait EI is a relatively stable disposition, but there is evidence showing that EI can be increased via programs targeting the core emotional competencies (identification, understanding, expression, regulation, and use of emotions), and that relatively short training initiatives (usually between 15 and 18 h) are already sufficient to produce a significant decrease in psychological distress with a corollary significant increase in well-being and health (Nelis et al., 2009; Kotsou et al., 2011). The effects of these EI trainings are not only statistically significant but also practically meaningful. For instance, in an elegant controlled trial, Karahan and Yalçin (2009) demonstrated that their 18-h EI training course (comprising 12 one-and-a-half hour sessions) reduced burnout by approximately 50%. For a review of the most robust studies on this issue, see Kotsou et al., 2018).

Furthermore, nurses may need practical training improving their ability in using adaptive emotion regulation strategies that help them to cope with emotion-laden situations and reduce stress responses (Roger and Hudson, 1995; Meichenbaum, 2007). During such training particular emphasis should be placed on providing nurses with knowledge of the effectiveness of various emotion regulation strategies. Research shows that people differ substantially with respect to emotion-related knowledge (Wranik et al., 2007; Mikolajczak, 2009) and some nurses may simply not know how to cope with emotionally demanding situations and how to reduce distress associated with NE.

It is likely, however, that the effect of such training would be lower among nurses who face many risk factors (not only emotionally demanding situations and daily work hassles but also exclusion from decision-making process and inadequate management). Therefore, the EI training should be supplemented with activities improving the organization of work and people management. Last but not least, according to a proverb, “prevention is better than cure,” programs providing EI training should be included in advance in nursing education. Such training would be beneficial for future nurses not only because it would help them to reduce the likelihood of job burnout, but also because EI skills increase the likelihood of successful completion of nursing education (Snowden et al., 2018).

There are several limitations to the current study that should be acknowledged. First, our data relied exclusively on self-report instruments, which could lead to concerns about common method variance (Podsakoff et al., 2003). We, however, assessed predictors (NE and trait EI) and outcome variables (burnout) at different points in time, which reduced the likelihood that our findings are solely due to common method variance. Moreover, emotions were measured in five sessions, which also ruled out the possibility that momentary affect drove the significant relationship between NE and burnout. Finally, we controlled for trait affectivity, which constituted a more conservative test of the emotions–burnout relationship. Nevertheless, future studies might use additional sources of data, such as peer reports, to strengthen the findings. Second, although we had five emotion measurements, the design was not cross-lagged; hence, statements about causal relationships should be put forward with caution until these results are replicated in a cross-lagged research design. Third, the use of data from female nurses only prevents generalization to males and to other organizational settings. There is, however, some evidence coming from research conducted among service sector employees, that trait EI plays a protective role in the relationship between NE at work and burnout. Szczygieł and Bazińska (2013) demonstrated that shop assistants and banking customer service representatives who declared greater intensity of NE (e.g., irritation and anger) while interacting with clients reported more symptoms of emotional exhaustion; this effect, however, was observed only among low-trait-EI employees. It would be desirable for further research to be conducted in more diverse groups, both in terms of gender and types of organizations. Fourth, the study sample size was small, thus further larger research is needed to confirm the results described here. Fifth, future researchers may consider taking seniority as a nurse as a control variable to refine the results.

Despite the limitations noted above, we believe that our study deserves attention, as it demonstrates that NE experienced at work increase nurses’ vulnerability to job burnout, but also that EI mitigates this effect. Moreover, there is evidence suggesting that even if NE cannot be avoided in daily nursing work, EI training can help reduce their adverse manifestations (Nelis et al., 2009; Kotsou et al., 2011). The past decade has witnessed an abundance of theoretical and empirical work dealing with EI in the nursing profession (e.g., Gerits et al., 2005; Kooker et al., 2007; Landa et al., 2008; Quoidbach and Hansenne, 2009) but knowledge about the role of EI in nursing is still limited (Bulmer-Smith et al., 2009; Petrides and Sevdalis, 2010; McCloughen and Foster, 2018). We believe that our research contributes to this under-researched field and provides inspiration for future research.

## Ethics Statement

All study procedures were approved by the Ethics Committee of the SWPS University of Social Sciences and Humanities (Poland), WKE-S-16-V-8. All subjects gave written informed consent in accordance with the Declaration of Helsinki.

## Author Contributions

DS developed the study design, performed the data collection, and analyzed the data. DS and MM contributed to data interpretation and writing the manuscript and approved the final version of the manuscript for submission.

## Conflict of Interest Statement

The authors declare that the research was conducted in the absence of any commercial or financial relationships that could be construed as a potential conflict of interest. The handling Editor declared a past collaboration with one of the authors MM.
